# Are They Just Two Children COVID-19 Cases Confused With Flu?

**DOI:** 10.3389/fped.2020.00341

**Published:** 2020-06-05

**Authors:** Biao Zou, Di Ma, Yinhu Li, Liru Qiu, Yu Chen, Yan Hao, Xiaoping Luo, Sainan Shu

**Affiliations:** ^1^Pediatric Department, Tongji Hospital, Tongji Medical College, Huazhong University of Science and Technology, Wuhan, China; ^2^Department of Computer Science, City University of Hong Kong, Hong Kong, China

**Keywords:** COVID-19, children, influenza, serum antibody of SARS-CoV-2, nucleic acid

## Abstract

COVID-19, an emerging infectious disease, has quickly spread all over the world. All human populations are susceptible to this disease. Here we present two pediatric COVID-19 cases, both of whom exhibited negative SARS-CoV-2 nucleic acid tests upon nasopharyngeal swab and were initially diagnosed with influenza A infection. COVID-19 was later confirmed in both patients by serum antibodies of SARS-CoV-2 and nucleic acid test on stool samples. Because children are susceptible to many respiratory pathogens, especially influenza, we concluded that children can be coinfected with multiple pathogens, and more attention should be paid to the exploration of SARS-CoV-2 during the pandemic of COVID-19. This report shows the possibility of misdiagnosis or missed diagnosis of children with COVID-19. We suggest that highly suspected pediatric COVID-19 cases with negative nucleic acid tests on nasopharyngeal swabs should be further checked by performing a nucleic acid test on stool samples and testing serum for antibodies against SARS-CoV-2.

## Introduction

Since December 2019, the outbreak of severe acute respiratory syndrome coronavirus-2 (SARS-CoV-2) has spread globally (1–3). On February 28 2020, WHO has declared COVID-19 as pandemic. Children are just as likely as adults to be infected with the new coronavirus, although their symptoms tend to be mild. According to Niccolo Parri's research, some of the COVID-19 children are even asymptomatic, yet rare cases of severe of critical disease are still reported (4). Several papers reported that every stage of childhood, including even neonates, are susceptible to the virus (5, 6). Recent studies also showed that attack rates for different age groups were similar. For example, the rate of infection in children under 10 was 7.4%, which is very similar to the rate of infection in adults of 7.9% (7).

Nucleic acid tests of respiratory samples are one of the essential conditions of diagnosis. The false-negative rate has been relatively high (8), so there is an urgent need to develop more sensitive and efficient complementary methods (9). Moreover, flu season usually dominates winter and spring, which confuses efforts to diagnose, and the effect of influenza infection on the diagnosis of COVID-19 remains unclear. Here, we report two pediatric COVID-19 cases initially misdiagnosed as influenza.

## Case 1

**Figure 1:**
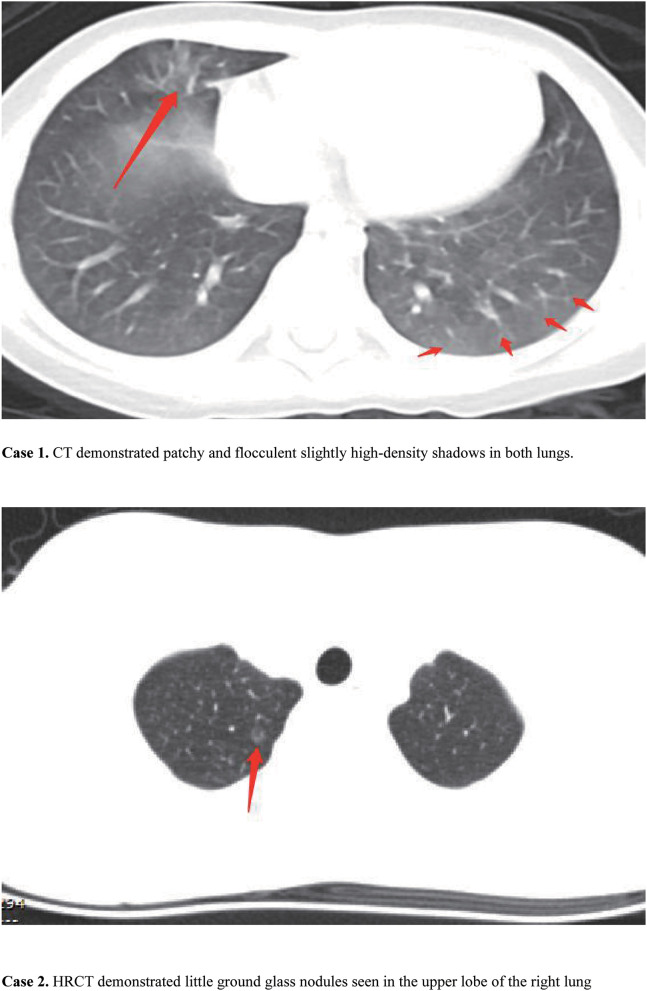
Chest computed tomography (CT) for the two cases. In case 1, CT demonstrated patchy and flocculent slightly high-density shadows in both lungs. In case 2, HRCT demonstrated little ground glass nodules seen in the upper lobe of the right lung.

On February 5, 2020, a 28-month old girl from the Wuhan urban area was referred to our hospital for intermittent fever that had lasted for 6 days (Figure 1). Her temperature peak was 39°C, accompanied with mild dry-cough. Before coming to our hospital, the child had been treated with the oral medications Tamiflu and Cefaclor for 3 days. Her paternal aunt was a suspected case of COVID-19. She had developed symptoms on January 26, 2020 and died on February 1. Neither the girl nor any other member of the family, including her parents, grandparents, and elder sister, had contact with her aunt in the previous month. No other members of the family had any symptoms.

**Figure 2:**
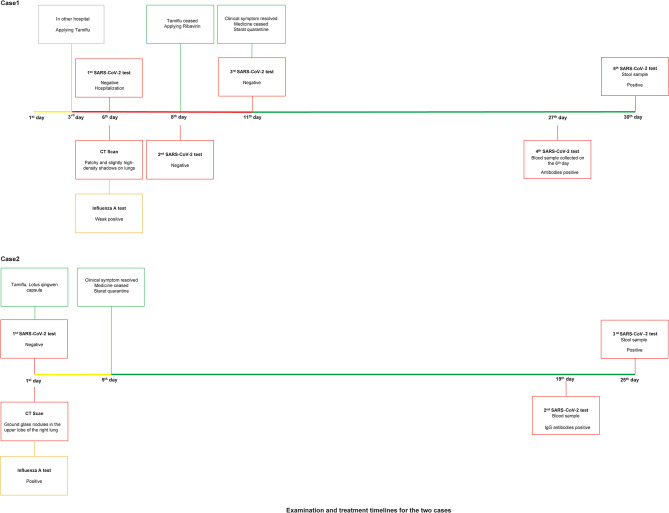
Examination and treatment timelines for the two cases. Lines in different colors represent different clinical symptoms: yellow line stands for fever, red line stands for fever and dry cough, green line stands for quarantine.

The results of her physical examination were as follows: temperature 38.8°C, pulse 110 beats per minute, respiration 25 breaths per minute, SpO_2_ 100%. No abnormal respiratory signs were observed. Nasopharyngeal swab samples were negative for SARS-CoV-2 on February 5. The serum IgM antibody of influenza A was weakly positive. Chest computed tomography (CT) showed patchy and flocculent slightly high-density shadows in both lungs (Figure 2). Blood Routine results included a leukocyte count of 9.29 × 10^9^/L and lymphocytes of 1.63 × 10^9^/L. C-reactive protein was 5 mg/L (Table 1).

**Table 1:** Results of Laboratory examinations.

	**Case 1**	**Case 2**	**Normal range**
Leukocytes (× 109/L)	5.52	7.83	4–12 for case1 3.5–9.5 for case2
Neutrophils (× 109/L)	3.43	5.61	1.5–8.5 for case1 1.8–6.3 for case2
Lymphocytes (× 109/L)	1.42	1.62	1.5–7 for case1 1.5–3.2 for case2
Hemoglobin (g/L)	136	146	110–147
Platelet (109/L)	148	223	125–350
Reactive protein (mg/L)	0.9	0.1	0–10
D—D dimer	0.22	0.22	0–0.5
Alanine aminotransferase (U/L)	9	18	≤ 33
Aspartate aminotransferase (U/L)	26	21	≤ 32
Influenza A, influenza B, mycoplasma pneumoniae, chlamydia, parainfluenza virus, adenovirus, respiratory syncytial virus, legionella pneumophila	Influenza A, weakly positive (day of illness); mycoplasma pneumonia, uncertain, others all negative	Influenza A was positive, others all negative	All negative
Nasopharyngeal swab nucleic acid	Negative (On February 5th, 7th, 10th)	Negative (On February 7th)	Negative
Fecal nucleic acid	Positive (30 day of illness)	Positive (25 day of illness)	Negative
Serum SARS-CoV-2 IgM	Positive (6 day of illness) 669.85 IU/ml	Negative (19 day of illness) 4.99 IU/ml	≤ 10 IU/ml
Serum SARS-CoV-2 IgG	Positive (6 day of illness) 244.22 IU/ml	Positive (19 day of illness) 181.19 IU/ml	≤ 10 IU/ml

Based on the findings given above, the girl was considered a case of influenza A virus infection and she was suggested to be isolated and treated with Tamiflu continuously. By February 7, however, fever had not yet abated. A second nasopharyngeal swab sample was taken and tested for SARS-CoV-2 again, but the result was still negative. On February 10, the girl's temperature returned to normal. The third nasopharyngeal swab sample was also negative. In late February, kits for the SARS-CoV-2 antibody (chemiluminescence assay) test became available. Serum collected on February 5, 2020 (the 6th day after the onset of disease) was found to be positive for both IgG and IgM antibodies against SARS-CoV-2. We collected a stool sample on February 29, and the nucleic acid test for SARS-CoV-2 was a strong positive. Finally, this child was confirmed to be a case of COVID-19 along with influenza A virus infection.

## Case 2

A 13-year-old boy came to a pediatric fever clinic in our hospital with intermittent fever having lasted 1 day on February 7, 2020 (Figure 1). His mother was a suspected case of COVID-19. She developed a fever on February 1, 2020. Her CT showed a few signs of infection. She was hospitalized in Wuhan Central Hospital for 4 days, and the SARS-CoV-2 nucleic acid tests were negative twice. The boy had been in close contact with his mother.

Physical examination showed: temperature 37.4°C, pulse 88 beats per minute, respiration 23 breaths per minute, SpO_2_ 100%. No abnormal respiratory signs were found. Nasopharyngeal swab samples were collected and tested negative for SARS-CoV-2. The serum IgM antibody of Influenza A was positive. A high-resolution chest computed tomography (HRCT) scan on February 7 demonstrated little ground glass nodules seen in the upper lobe of the right lung (Figure 2). Other laboratory findings included a leukocyte count of 7.83 × 109/L, lymphocytes of 1.62 × 109/L, and C-reactive protein of < 0.1 mg/L (Table 1).

Based on the results of lab examination, the boy was also considered an influenza case, although COVID-19 could not be ruled out. Isolation treatment was recommended. Then, he started treatment with Tamiflu and lotus qingwen capsules. Five days later, his temperature was normal. Considering the boy's and his mother's medical history, serum antibody of SARS-CoV-2 and fecal nucleic acid were assessed on February 27. The subsequent results for both were positive, so this boy was also confirmed to be a COVID-19 case complicated with influenza A virus infection.

## Discussion

Here we reported two pediatric COVID-19 cases who were initially diagnosed as influenza A infection, but COVID-19 could not be ruled out due to their abnormal lung images and medical history and the high false negative ratio of SARS-CoV-2 nucleic acid results of upper-respiratory samples.

Both cases came from Wuhan, and case 2 had a history of close contact with suspected COVID-19 patients. They exhibited mild to high fever, and their CT scans showed mild lung shallow lesions. They also both had slightly low lymphocyte counts. All of the evidence cited here indicated that we should assess the patients for signs of COVID-19, which might coexist in parallel with the influenza A infection. For this reason, we performed SARS-Cov-2 IgM and IgG antibody tests on serum and nucleic acid examinations on fecal samples.

The negative results of previous pharyngeal swabs may have any of the following several causes. First, poor coordination in children often affects the quality of the swabs. Second, the technicians may have had limited collection skills. Without adequate training and concern about the risk of infection, medical professions might not collect the samples from the right place with the swabs. Last but not least, sampling time has a direct bearing on the positivity rate. After the acute phase of COVID-19, the positivity rate of pharyngeal swabs dropped rapidly (10). Blood and rectal swabs and fecal samples have several advantages compared with nasopharyngeal swabs. First, it is more convenient to conduct sampling and there is less need for the child's cooperation and the professional skills on the part of the person collecting the sample. Antibodies and nucleic acids persist longer in serum and feces than in nasal swabs (11). Several papers have reported a significant lag time in the detection of viral RNA of SARS-CoV-2 in patient feces, even occurring during the recovery period (5, 12).

Furthermore, the symptoms of pediatric COVID-19 are largely non-specific. In addition to CT imaging and lymphocyte counts different from those of adults, children with COVID-19 are susceptible to other etiological infections, and we should be aware of possible coinfections. In a previous study, we retrospectively detected nasopharyngeal swab samples of 366 hospitalized children during January 7 to January 13. The results showed that influenza A and B were the top two viral pathogens, with 23 and 20 cases respectively, while SARS-CoV-2 was detected in 6 patients (about 1.6 percent) (13). Among those 6 patients, 3 children were identified with additional infection of Mycoplasma pneumoniae or Legionella pneumophila. Therefore, as one of the most common respiratory pathogen, influenza viruses should not be ignored despite the prominence of the COVID-19 pandemic. Secondly, children are prone to multiple respiratory pathogens, which might lead to missed diagnosis and misdiagnosis. The number of COVID-19 children might be underestimated due to their non-specific manifestations, less medical attention and detection, more positive detection of other pathogens, relatively higher false negative rate of nucleic acid detection, and so on. All these factors contribute to the transmission of SARS-CoV-2 and do harm to disease control (14).

From these two cases, we suggest that specific antibody tests and fecal nucleic acid detection for SARS-CoV-2 should be used as a complementary method for pediatric patients in whom there are strong reasons to suspect COVID-19, especially when respiratory samples are negative or the time of testing has exceeded the acute phase of the disease. This can prevent missed diagnosis or misdiagnosis. The impact of viral nucleic acid in feces on disease transmission should be assessed further as well.

## Data Availability Statement

All datasets presented in this study are included in the article/supplementary files.

## Ethics Statement

The studies involving human participants were reviewed and approved by Human Ethics Committee of Huazhong University of Science and Technology. Written informed consent to participate in this study was provided by the participants' legal guardian/next of kin. Written informed consent was obtained from the individual(s), and minor(s)' legal guardian/next of kin, for the publication of any potentially identifiable images or data included in this article.

## Author Contributions

SS and XL conceptualized and designed the study, drafted the initial manuscript, and reviewed and revised the manuscript. BZ, DM, and YL collected data, carried out the initial analyses, drafted the initial manuscript, and reviewed and revised the manuscript. LQ, YC, and YH designed the data collection instruments, coordinated and supervised data collection, and critically reviewed the manuscript. All authors approved the final manuscript as submitted and agreed to be accountable for all aspects of the work.

## Conflict of Interest

The authors declare that the research was conducted in the absence of any commercial or financial relationships that could be construed as a potential conflict of interest.
